# Cancer epigenetics: Moving forward

**DOI:** 10.1371/journal.pgen.1007362

**Published:** 2018-06-07

**Authors:** Angela Nebbioso, Francesco Paolo Tambaro, Carmela Dell’Aversana, Lucia Altucci

**Affiliations:** 1 Dipartimento di Medicina di Precisione, Università degli Studi della Campania “L. Vanvitelli,” Napoli, Italy; 2 Struttura Semplice Dipartimentale Trapianto di Midollo Osseo-Azienda Ospedialiera di Rilievo Nazionale, Santobono-Pausilipon, Napoli, Italy; Albert Einstein College of Medicine, UNITED STATES

## Abstract

Defects in chromatin modifiers and remodelers have been described both for hematological and solid malignancies, corroborating and strengthening the role of epigenetic aberrations in the etiology of cancer. Furthermore, epigenetic marks—DNA methylation, histone modifications, chromatin remodeling, and microRNA—can be considered potential markers of cancer development and progression. Here, we review whether altered epigenetic landscapes are merely a consequence of chromatin modifier/remodeler aberrations or a hallmark of cancer etiology. We critically evaluate current knowledge on causal epigenetic aberrations and examine to what extent the prioritization of (epi)genetic deregulations can be assessed in cancer as some type of genetic lesion characterizing solid cancer progression. We also discuss the multiple challenges in developing compounds targeting epigenetic enzymes (named epidrugs) for epigenetic-based therapies. The implementation of acquired knowledge of epigenetic biomarkers for patient stratification, together with the development of next-generation epidrugs and predictive models, will take our understanding and use of cancer epigenetics in diagnosis, prognosis, and treatment of cancer patients to a new level.

## Introduction

Although the complete sequence of the 3 billion base pairs that make up the human genome has been generated thousands of times [1,2], identifying genomic variations across the cell types that contribute to health and disease remains a major challenge.

**Fig 1:**
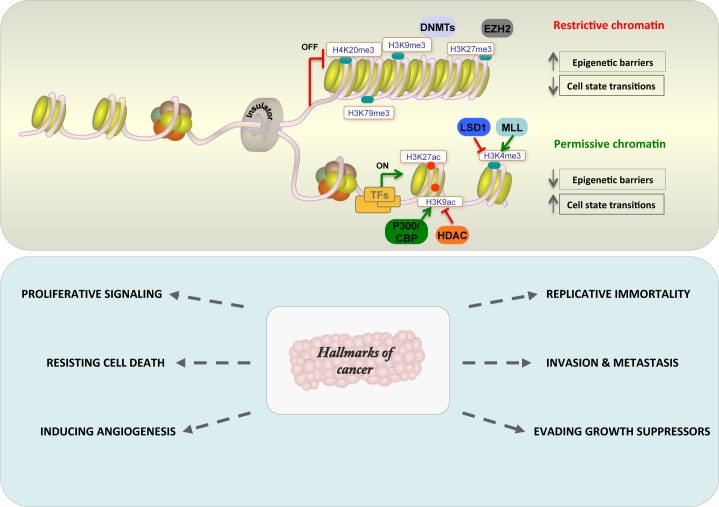
Chromatin structure determines gene expression and hallmarks of cancer. (A) Chromatin can assume active and repressive states. Repressive states are supercoiled and enriched for DNA and histone methylation marks; active states are accessible to transcription factors (TFs) and enriched for histone marks (such as H3K27ac and H3K4me3). Restrictive chromatin raises epigenetic barriers and blocks cell state transition, while permissive chromatin reduces epigenetic barriers and determines alternate cell states. (B) Aberrant permissive and restrictive chromatin states cause cancerogenesis and give rise to hallmarks of cancer.

In 1942, Conrad Waddington coined the term “epigenetics” to describe inherited changes in phenotype without changes in genotype [3,4]. In the current view, the meaning of epigenetics has become more comprehensive, often specifying a “stably heritable phenotype resulting from changes in a chromosome without alterations in the DNA sequence” (2008 Cold Spring Harbor Epigenetics meeting). In Waddington’s developmental landscape, differentiating cells are “canalized” by specific environmental stimuli to follow different routes or canyons separated by mountain walls. The height of the walls increases during differentiation, symbolizing progressive loss of multi-potency and lineage restriction. Epigenetic and transcriptional regulators work in concert “adjusting the height of the walls” to restrict cells to a particular canyon, so that mature cells display different phenotypes even though they started off with the same genotype. Transcriptional and epigenetic regulations have emerged as key players in determining normal physiology and cell type identities [5]. Endogenous and exogenous stimuli can deviate “the trajectory of cells,” reorganizing the chromatin structure, and thus, leading to aberrant gene expression or repression, allowing them to acquire the full set of so-called “cancer hallmarks” [6] (Fig 1). The reversibility of these alterations by epigenetic therapies has far-reaching implications for clinical prevention and treatment. Consequently, the need for reference epigenome maps of healthy and diseased cell types to study the effects of compounds on epigenetic enzymes and factors (epi-treatments) is evident. Enormous advances have been made in our understanding of how genetic and epigenetic mechanisms regulate physiological and pathological gene expression by global projects, such as the Encyclopedia of DNA Elements (ENCODE, 2003), The Cancer Genome Atlas (TCGA, 2006), the International Cancer Genome Consortium (ICGC, 2008), the National Institutes of Health Roadmap Epigenomics Mapping Consortium (2008), and the European Community initiative BLUEPRINT (2011). By applying next-generation sequencing-based approaches, these projects revealed epigenomic profiles in both healthy and pathological conditions. Epigenomic profiling has greatly enhanced our understanding of complex human diseases, including cancer. The International Human Epigenome Consortium (IHEC, 2010) [7] was founded to coordinate international efforts with the aim of producing reference maps of at least 1,000 epigenomes for key cellular states relevant to health and disease [8] and to disseminate data to improve clinical applications. In 2012, ENCODE annotated functional elements within the entire genome, identifying regions of transcription, transcription factor (TF) association, chromatin structure, and histone modification in 147 different cell types [9]. In 2015, the Roadmap Consortium extended ENCODE findings, clarifying the role of epigenetic mechanisms in human biology and disease [10] and creating a publicly accessible collection of 127 human epigenomes. Roadmap scientists matched this epigenomic dataset to trait- and disease-associated variants identified by genome-wide association studies (GWAS). These genetic variants are frequently enriched in tissue-specific epi-marks (specifically, H3K4me1-marked active/poised enhancers), underscoring the importance of particular cell types for discrete human traits. Their results indicate that enhancer-associated marks are informative for tissue-specific enrichments for regulatory regions, but that promoter-, open chromatin- and transcription-associated marks are also informative for other significant enrichments, suggesting the pleiotropic role of disease variants.

The scientific achievements of IHEC, partially released as a package in Cell and Cell Press-associated journals (http://www.cell.com/consortium/IHEC), together provide a greater understanding of epi-marks on the human genome that may prove useful in diagnosis and therapy of human diseases. Here, we summarize the findings from this collection of data from the perspective of how they have enhanced our knowledge of the pathogenesis of human cancers and their importance as prognostic and therapeutic markers. Since the number of cancers and cancer-related data is constantly increasing, we focus on specific examples of normal hematopoiesis, as well as hematological malignancies, breast cancer (BC) and rhabdoid tumors. The common denominator of all these cancer types is that, in addition to genome deregulation, deregulated epigenomic features have been determined and innovative treatments using chromatin-targeting drugs have been proposed or are in preclinical trials.

## Transcription factors and chromatin crosstalk in tumorigenesis

The transcriptional expression of a given locus is to a large extent determined by chromatin conformation: the region must be accessible to regulatory factors and transcriptional machinery. The homeostatic chromatin network is determined by the close interplay between polycomb family repressors, trithorax family activators, and chromatin remodelers [11] (Fig 1). Mutations in genes coding for these factors impact strongly on epigenetic homeostasis, and deregulation can lead to tumorigenesis. For example, active loci are associated with TFs and chromatin modifiers such as KDM, p300, ARID1A/B, and MLL components, which trigger transcriptional activity. Mutations in MLL1 and CBP/p300 block correct commitment of regulatory regions in leukemia. In particular, leukemia-associated translocations involving MLL1 generate fusion protein-driven malignant transformation, which is controlled by HOX family genes and the HOX cofactor MEIS1 [12]. Further, gain-of-function *EZH2* mutations were found in several lymphomas, characterized by aberrant histone H3 trimethylation at amino acid position 27 (H3K27me3) that block B-cell development [13] due to repression of lineage-specific developmental B-cell genes [14,15]. Repressive states of chromatin can persist throughout cell divisions by the action of specific histone modifications, DNA methylation, regulatory proteins, and non-coding RNA [16]. Active chromatin-remodeling enzymes are inactive in many human cancers, promoting global chromatin restriction. However, in many neoplasias, the aberrant CpG island methylator phenotype (CIMP) can suppress tumor suppression genes (TSGs) such as *p16*, as well as DNA mismatch repair genes, including *MLH1* and *MSH2* [17]. DNA hypermethylation reduces binding of the transcriptional repressor CTCF, causing insulator dysfunction frequent in Isocitrate Dehydrogenase (IDH) mutant gliomas [18]. IDH mutants have highlighted the tight crosstalk between epigenetics and metabolism via the formation of a so-called “oncometabolite,” 2-hydroxyglutarate, which is a competitive inhibitor of α-ketoglutarate(KG)–dependent enzymes, such as TET2, leading to DNA and histone hypermethylation and a differentiation block [19]. Thus, the response of a locus to stimuli depends on the expression and binding of specific TFs to regulatory regions. Once bound to DNA, TFs can also modify the chromatin landscape, recruiting chromatin modifying and remodeling enzymes. In this scenario, TFs such as RUNX1, RARα, and CBFB (and their oncogenic derivatives) provide clear examples of transcriptional and epigenetic reprogramming driving leukemogenesis [20]. Different cues can result in aberrantly permissive or restrictive chromatin states that can lead to oncogene activation or tumor suppressor inactivation, enabling cells to acquire the six essential hallmarks of cancer [6] (Fig 1).

## Altered epigenetic landscapes: Consequence or hallmark(s) of human cancer?

The hallmarks of human cancer (proliferative signals, cell death impairment, inactivation of growth suppressors, angiogenesis, replicative independence leading to immortality, and acquirement of cancer progression features such as invasion and metastasis) were defined by Hanahan and Weinberg as the driving forces of tumorigenesis [6,21]. These hallmarks identified cancer as a disease of the genome. The fact that the classical hallmarks of human cancer can potentially be achieved “purely” through epigenome deregulation [18] questions the current view of tumorigenesis, suggesting that epigenome deregulation may cause cancer without genetic contribution.

Aberrant proliferation bursts can, for example, occur by insulator loss, tumor suppressor silencing may be associated to DNA hypermethylation or hyperactivity of EZH2, immortality can be sustained by non-genetic self-renewal of the stem cell state, angiogenesis can be modulated by VHL promoter methylation, cell death impairment may be linked to modification of the epigenetic state of apoptotic players, and invasion and metastasis may be mediated by cell state transitions such as (but not restricted to) epithelial-mesenchymal transition [18]. Whether and how frequently “purely” epigenome-mediated non-genetic causal events co-occur in tumorigenesis can, currently, only be speculated. The best example of a tumor potentially driven by epigenetic forces (though still with a genetic lesion) is small cell carcinoma of the ovary, hypercalcemic type [22], invariably characterized by a single coding mutation in *SMARCA4* together with genome-wide deregulation of methylation. However, it is important to note that our understanding of epigenome alterations in cancer is at the very early stages. Our insight into how epigenetic deregulations trigger tumorigenesis is in no way comparable to the body of knowledge we have built up on cancer genome alterations. In addition, the stages preceding clinically evident cancer are affected by a number of interacting factors that can be grouped into genetic, epigenome-based and environmental factors, commonly referred to as “predisposition.” While the contribution of hereditary epigenetic deregulations to cancer has been clarified, whether (and how) tumorigenesis may be a purely epigenome-based multi-step process remains to be defined.

New single-cell technologies may profoundly change our view and better distinguish between genetic vs epigenetic driver and passenger events [23]. The application of single-cell transcriptomics is likely to unveil the variability and heterogeneity of cancer cells and of “normal cells” positioned in cancer tissue and premalignant lesions. Assays devoted to decrypting dynamics and temporal resolution [24,25] might also explain whether and which stochastic epigenetic deregulations in cells represent an early indicator of tumor aggressiveness and to what extent transition states and rapid changes play a role. Similarly, new 3D models of tumorigenesis, such as organoids and spherical models, will be required to clarify the potential molecular relationship between tumor cells, microenvironment, and position of cancer cells within the tissue, since current models are based on the paradigm that cancer is a genetic disease.

Cancer-related epigenome aberrations now being observed might reside in both 1) genetic mutations of chromatin modifiers and remodelers [26,27] and 2) non-genetic deregulations. Chromatin modifiers use metabolites as cofactors and are sensitive to minor changes in their balance [28,29]. Nutrition, metabolic states, interplay with (tumor)-microenvironment, and ageing may, thus, represent examples of tumorigenic epigenetic alterations instigated by non-genomic stimuli. These findings open up several considerations.

The availability of donors and cofactors (such as vitamin C, folates, acetyl-CoA, SAM, α-KG) influences cell decision-making and might contribute to restriction point control, suggesting their intriguing potential as a therapeutic intervention point [28].

A better knowledge of genome-epigenome interplay is crucial. It is now quite clear that the integration of crosstalk between genome and epigenome may pave the way to advanced novel diagnostic and prognostic tools, as well as treatments. It is worth underlining that epigenome deregulation will likely have an impact on chromatin as a whole, indicating that a multidimensional concept of cancer-driving events might also be based on 3D cancer cell deregulation. New therapies should take this concept into account.

## Normal hematopoiesis and hematological malignancies

The role of genetic and epigenetic alterations in cancer was initially discovered in hematological malignancies, heterogeneous disorders characterized by arrest of differentiation and uncontrolled proliferation of hematopoietic stem cells (HSCs). Gene expression and genome-wide DNA methylation profiles have become precise tools for cell type prediction throughout hematopoietic lineage in health and disease. By clarifying the role of epi-modifications in hematopoiesis, a significant step forward in precision medicine for blood cancers has been made.

BLUEPRINT highlighted new epi-dynamics in hematopoiesis. Farlik and colleagues produced genome-wide reference maps of DNA methylation dynamics in HSC differentiation [30]. Cell type-specific DNA methylation patterns were profiled in a meta-epigenomic analysis. HSCs and progenitor cells displayed a similar distribution of DNA methylation levels, while differentiated myeloid cells exhibited the lowest values. HSCs and multi-potent progenitors derived from different sources presented different DNA methylation profiles, with lower levels found in peripheral blood cells. Additionally, hypomethylated regions in HSCs from peripheral blood overlapped binding sites of CTCF, the cohesin complex, and the TFs RUNX3 and ZNF143. DNA methylation reduction at important TF binding sites marked commitment of precursors towards myeloid lineage. DNA methylation levels at regulatory regions were on average reduced in myeloid cells compared to lymphoid cells. Binding sites for 11 TFs and RNA polymerase II were enriched in differentially methylated regions in myeloid and lymphoid precursors. Consistently, DNA methylation variations were also associated with histone modifications; open chromatin was associated with H3K4me1, and active enhancer-linked H3K27ac was observed in myeloid and lymphoid cells, respectively. From this epigenome-wide analysis, the authors established a data-driven model of human hematopoietic development.

To investigate epigenetic mechanisms driving cell identity in hematopoietic differentiation, Schuyler and colleagues integrated 112 whole-genome bisulfite sequencing (WGBS) maps with CTCF-binding data and nucleosome occupancy, following the development of myeloid and lymphoid cells [31]. Genome-wide DNA methylation trends were distinct. Methylation levels remained relatively stable across myeloid lineage, but declined progressively during lymphoid maturation, reaching the lowest values in long-lived lymphocytes. Methylation outside of a CpG dinucleotide (mCH, with H standing for adenine, cytosine, or thymine) reached high and diffuse levels in all naïve T cell samples, and in uncommitted hematopoietic progenitor cells, intermediate levels in macrophages, and low levels in T-lymphocytes. Myeloid-derived leukemias, such as acute promyelocytic leukemia and acute myeloid leukemia (AML), showed substantial gains in mCH, while lymphoid-derived diseases, including mantle cell lymphoma (MCL), chronic lymphocytic leukemia (CLL), and multiple myeloma (MM), revealed diverse mCH levels. Oscillation of methylation levels was inverse to those of nucleosome occupancy and dependent on constitutively occupied CTCF-binding sites (cCTCFs). The amplitude of oscillating methylation trends increased across B-lymphocyte differentiation. Both B and T cells showed increased amplitude of oscillating methylation patterns at cCTCFs across lymphocyte development, while global average methylation levels decreased. A progressive shift of methylation from nucleosome-associated DNA to linkers was observed along B-lymphocyte development. In myeloid cells, oscillating methylation trends at cCTCFs were constant. Lymphoid-derived cancers confirmed the shift of methylation from nucleosome to linkers, while myeloid-derived cancers showed an amplitude of methylation trends comparable to normal myeloid samples or decreased compared to global methylation.

In 2013, TCGA analyzed the genome of 200 adult cases of *de novo* AML, demonstrating the presence of at least one non-synonymous mutation in 44% of DNA methylation-related genes and 30% of chromatin-modifying genes [32]. This landmark finding demonstrated the involvement and cooperation of both mutations in genes encoding for epigenetic players and somatic genetic mutations in AML pathogenesis. Novel recurrent mutations including *ASXL1*, *TET2*, *IDH1/2*, and *DNMT3A* are useful as biomarkers for better prognostic and therapeutic risk stratification. By non-negative matrix factorization (NMF) consensus clustering, TCGA identified five miRNA-sequencing groups differently associated with French American British (FAB) classification AML subtypes and cytogenetic risk categories. For example, Group 3 was associated with a hallmark high- and low-expression of miRNA-10a and miRNA-424, respectively. By analyzing DNA methylation, significant differences were observed across AML samples at 42% of CpG loci examined, with 67% and 33% resulting in a gain and loss of methylation, respectively. Compared to healthy CD34^+^CD38^-^ cells, *IDH1/2* mutations showed extensive gains of DNA methylation, while samples with triple mutations in *NPM1/DNMT3A*/*FLT3* showed extensive loss of DNA methylation. Integrated analysis of these data revealed that a specific subtype of AML is associated with unique epigenetic features, such as specific miRNA and DNA methylation signatures. Recently, WGBS on these primary AML samples addressed the role of DNMT3A-dependent methylation in leukemogenesis. Specifically, the data demonstrated that hypomethylation is an initiating factor in AML harboring the DNMT3A^R882^ mutation, while DNMT3A-dependent hypermethylation is a consequence rather than a cause of AML development [33].

Queiros and colleagues unraveled the role of DNA methylation in driving development of chronic MCL across normal B-cell lineage [34]. By DNA methylome analysis of 82 MCLs, they identified two subtypes whose cells reflect epigenetic imprints of germinal-center–inexperienced and germinal-center–experienced B cells. DNA methylation profiles in malignant cells resulted, determined by methylation dynamics of normal B cells across maturation. An integrative analysis revealed several differentially methylated regions in regulatory elements, including a distant enhancer showing de novo looping to the MCL oncogene *SOX11*, whose expression correlates with poor outcome. These findings provided new insights into lymphomagenesis, allowing better patient risk stratification and possibly improved prognosis and treatments. The data corroborated previously published results in CLL [35,36], another B-cell blood cancer. Although few genetic alterations have been identified in CLL, extensive epigenomic deregulation is associated with clinical characteristics and patient subgroups [35,37,38].

Several leukemia subtypes derive from chromosomal translocations. To drive leukemogenesis, onco-fusion proteins use different molecular mechanisms, including epigenomic deregulation, as reported for PML-RARα in AML [39,40]. The translocation t(v;11q23) causes rearrangements of *KMT2A*, responsible for KMT2A-resistent (KMT2Ar) acute lymphoblastic leukemia (ALL). Bergmann and colleagues showed that array-based DNA methylation profiles of pediatric KMT2Ar ALLs significantly differed from those of normal B-cell precursors and other ALL subtypes [41]. Hyper- and hypomethylated CpG loci were enriched for the GO terms cell–cell signaling and GTPase signal transduction, defining a KMT2Ar ALL signature. Compared to B-cell precursors, KMT2Ar ALL cells displayed significantly hypomethylated CpGs at enhancer regions, including *HDAC4*, which is associated with poor prognosis [42], *DOT1L*, involved in the development of KTM2Ar ALL [43], and *MSI2*, which is important for the prediction of poor survival in all ALLs [44] and for maintaining MLL-driven self-renewal [45]. Further analysis correlated DNA methylation remodeling with TF binding; hypermethylated regions were enriched for *NANOG*, involved in pluripotency and self-renewal. These data further underscore the impact of epigenetic remodeling on leukemia-specific expression changes in KTM2Ar ALL.

The translocation t(8;21) generates AML1-ETO fusion protein, responsible for differentiation arrest and cell survival in 10% of AMLs. t(8;21) transforms the transcriptional activator RUNX1 into a repressor, inhibiting expression of RUNX1 target genes [46–49]. Increasing evidence indicates that disease outcome depends on complex molecular mechanisms. Mandoli and colleagues investigated molecular aspects of AML1-ETO in primary blasts and cell lines, determining the identity and role of each component within AML1-ETO complex [50]. AML1-ETO binding sites were found at both promoter and distal elements with specific chromatin characteristics; binding sites in promoters were highly acetylated and associated with gene expression, whereas distal sites showed reduced acetylation and low expression of associated genes. By comparing promoter and distal AML1-ETO complexes, they identified both similar and different hematopoietic, splicing, and chromatin-remodeling regulators. Leukemic maintenance appeared to be dependent on a fine balance between *AML1-ETO*, *RUNX1*, and *ERG* expression; knockdown of either *ERG* or *RUNX1*, both of which are up-regulated in AML1-ETO, resulted in cell death [51]. Cell death associated with overexpression of AML1-ETO in differentiated induced pluripotent stem cells showed that only a small amount of the protein is required for leukemogenesis, corroborating previous studies [52,53]. Anticancer therapies able to up-regulate *AML1-ETO* by altering its equilibrium with *RUNX1* and *ERG* may provide promising strategies targeting t(8;21) cells.

MM is a clonal B-cell disorder in which tumor plasma cells expand and accumulate in bone marrow and accounts for about 13% of hematological cancers. Complex and heterogeneous genome and epigenome deregulation is causal to MM. Deregulation of DNA methylation pathway contributes to disease initiation and progression [54,55]. CpG promoter hypermethylation leads to silencing of TSGs and/or miRNAs [56]. Abnormal histone methylation patterns, often initiated by overexpression of histone methyltransferases (HMTs), are observed and correlated with drug resistance [57].

Consistently, several DNMT and HMT inhibitors [58] and miRNAs are emerging as promising diagnostic and therapeutic tools for MM. Interestingly, the histone deacetylase inhibitor (HDACi) and anti-angiogenesis agent panobinostat recently entered the clinic for patients already treated with bortezomib and immunomodulating agents (NCT01023308), strengthening the use of epi-based strategies for MM treatment [59].

Ample evidence has shown the clinical benefits of epidrugs in several hematological diseases, leading to their FDA approval for treatment of myelodysplastic syndromes and cutaneous T-cell lymphomas (CTCLs). Furthermore, ongoing pre-clinical and clinical trials are assessing epidrugs alone or in combination with other pharmaceuticals [60] (Table 1). Several HDACi are in clinical trials for CTCL therapy. Besides vorinostat and romidepsin, the FDA approved four CTCL treatments in 2006 and 2009, respectively: belinostat is in phase II (NCT00274651), panobinostat in phase I/II/III (NCT00412997/NCT00699296/NCT00490776/NCT00425555), quisinostat in phase II (NCT01486277), and romidepsin and SHAPE (SHP-141) in phase I/II (NCT00007345/NCT02213861/NCT01433731) trials. Follicular and diffuse large B-cell lymphomas harboring activating mutations in the HMT *EZH2*, the enzymatic component of polycomb repressive complex 2 (PRC2) [61], are good candidates for EZH2-targeted therapies using pharmacological inhibitors (EZH2i), such as GSK126, already shown to have antiproliferative activity in vitro and in EZH2 mutant models in vivo [13]. Currently, three EZH2i are in clinical trials in patients affected by B-cell lymphomas: GSK126 (NCT02082977), tazemetostat [62] (NCT01897571), and CPI-1205 (NCT02395601).

**Table 1:** Clinical trials for multiple myeloma and lymphomas.

NCT number	Clinical trial	Condition	Drug	Phase	Sponsor
NCT01023308	Panobinostat or placebo with bortezomib and dexamethasone in patients with relapsed multiple myeloma (PANORAMA-1)	Multiple myeloma	Panobinostat and bortezomib	3	Novartis Pharmaceuticals
NCT00274651	A phase II clinical trial of PXD101 in patients with recurrent or refractory cutaneous and peripheral T-cell lymphomas (PXD101-CLN-6)	Cutaneous T-cell lymphoma Peripheral T-cell lymphoma Non-Hodgkin's lymphoma	Belinostat	2	Onxeo
NCT00412997	LBH589 in adult patients with advanced solid tumors or cutaneous T-cell lymphoma	Cutaneous T-cell lymphoma	Panobinostat	1	Novartis Pharmaceuticals
NCT00699296	Study of oral LBH589 in patients with cutaneous T-cell lymphoma and adult T-cell leukemia/lymphoma	Cutaneous T-cell lymphoma Adult T-cell leukemia/lymphoma	Panobinostat	2	Novartis Pharmaceuticals
NCT00490776	Study of oral LBH589 inadult patients with refractory/resistant cutaneous T-cell lymphoma	Cutaneous T-cell lymphoma	Panobinostat	2/3	Novartis Pharmaceuticals
NCT00425555	Study of oral LBH589 in adult patients with refractory cutaneous T-cell lymphoma	Cutaneous T-cell lymphoma	Panobinostat	2/3	Novartis Pharmaceuticals
NCT01486277	A study of the histone deacetylase Inhibitor (HDACi) quisinostat (JNJ-26481585) in patients with previously treated stage Ib-IVa cutaneous T-cell lymphoma	Cutaneous T-cell lymphoma	Quisinostat, 12 mg	2	Janssen Research & Development, LLC
NCT00007345	Depsipeptide to treat patients with cutaneous T-cell lymphoma and peripheral T-cell lymphoma	Cutaneous T-cell lymphoma Peripheral T-cell lymphoma	Romidepsin	2	National Cancer Institute
NCT01433731	Safety, pharmacodynamics, pharmacokinetics study of SHP141 in IA, IB, or IIA cutaneous T-cell lymphoma	Cutaneous T-cell lymphoma	SHAPE	1	TetraLogic Pharmaceuticals
NCT02213861	Efficacy, safety and tolerability study of SHAPE in IA, IB or IIA cutaneous T-cell lymphoma	Cutaneous T-cell lymphoma	SHAPE	2	TetraLogic Pharmaceuticals
NCT02082977	A study to investigate the safety, pharmacokinetics, pharmacodynamics and clinical activity of GSK2816126 in subjects with relapsed/refractory diffuse large B-cell lymphoma, transformed follicular lymphoma, other non-Hodgkin's lymphomas, solid tumors and multiple myeloma	Cancer	GSK2816126	1	GlaxoSmithKline
NCT01897571	Open-label, multicenter, phase 1/2 study of Tazemetostat (EZH2 Histone Methyl Transferase [HMT] Inhibitor) as a single agent in subjects with adv. solid tumors or with B-cell lymphomas and tazemetostat in combination with prednisolone in subjects with DLBCL	B-cell lymphomas (phase 1) Advanced solid tumors (phase1) Diffuse large B-cell lymphoma (phase 2) Follicular lymphoma (phase 2) Transformed follicular lymphoma Primary mediastinal large B-cell lymphoma	Tazemetostat	1/2	Epizyme
NCT02395601	A study evaluating CPI-1205 in patients with B-cell lymphomas	B-cell lymphoma	CPI-1205	1	Constellation Pharmaceuticals

## Epigenetics in solid cancers

### Breast cancer

BC is a heterogeneous group of tumors, with each group having a unique prognosis and sensitivity to therapy. Although current treatments have reduced BC mortality, it remains one of the most common causes of cancer death among women worldwide [63]. Understanding the molecular and cellular mechanisms of tumor heterogeneity that are relevant to the diagnosis, prognosis, and therapy of BC is the subject of intense research. Tumor size, histological subtype and grade, lymph node status and expression of estrogen receptor (ER), progesterone receptor (PR), and human epidermal growth factor receptor 2 (HER2) are routinely used for classification of BC. A growing body of molecular data derived from cutting-edge genomic, transcriptomic, and epigenomic techniques has provided information that increases the accuracy of BC subtyping and generated prognostic and predictive classifiers for precision medicine. In 2012, TCGA reported the integrated genome-wide analysis of 466 primary BCs and demonstrated that genetic and epigenetic alterations converge phenotypically into four main disease subtypes showing molecular heterogeneity: luminal-A, luminal-B, basal-like, and HER2-enriched (HER2E) [64]. TCGA identified novel mutated genes involved in BC, found to be markedly more assorted and frequent within luminal-A/B subgroups; although, overall mutation rate was highest in basal-like and HER2E subtypes. TCGA also identified five DNA methylation clusters. Group 3, consisting predominantly of luminal-B subtype, displayed the highest levels of DNA methylation, fewer *PIK3CA*, *MAP3K1* and *MAP2K4* mutations, and lower expression of Wnt pathway genes. Group 5, comprised of mainly basal-like tumors, showed the lowest levels of DNA methylation and the highest frequency of *TP53* mutations. HER2-positive clinical status and HER2E mRNA subtype associated modestly with the methylation subtypes. DNA methylation and expression analyses comparing Group 3 with the other groups uncovered a set of 490 genes both methylated and down-regulated in Group 3 tumors, associated with “Extracellular region part” and “Wnt signaling pathway” functional annotations. In summary, TCGA identified many subtype-specific mutations that drive tumor biology and are therapeutically tractable.

Furthermore, using TCGA methylation data, 17 individual differentially methylated regions stratifying triple-negative BC (TNBC) patients into good and poor prognosis groups were identified [65]. Deregulation in gene-specific methylation in BC clinical development and therapy resistance was also well demonstrated. Interestingly, *ESR1* and *ARHI* methylation were good survival predictors in tamoxifen-treated and non-tamoxifen-treated patients, respectively. TNBC showed an enrichment of alterations in DNA repair genes, mainly BRCA1. BRCA1 promoter hypermethylation affected sensitivity to DNA-damaging chemotherapeutic agents, such as cisplatin [66] and to poly(ADP-ribose) polymerase inhibitors [67]. Hypermethylation-mediated silencing of several genes encoding Wnt-negative regulators WIF1 and DKK3 [68], TWIST, RASSF1A, CCND2, HIN1, CDH13, CDH1, and GSTP1 was also reported [69,70]. Finally, in a cohort of 70 primary TNBC samples, novel methylation changes of specific genes in primary tumors and lymph node metastases were defined [71]. Distinct BC subtypes showed unique epigenetic patterns and marks [72]. The relationship between high levels of H3K9ac and better disease-free survival and metastatic-specific survival was reported [73]. H3k9ac enrichment characterized HER2-positive and TNBC tumors [74]. H3K27me3 marks were diminished in luminal-B, HER2-positive, and TNBC tumors, but higher in the luminal-A subgroup [75]. Reductions in H3K9me2 and H3K9me3 with increased expression of the histone demethylase KDM3A (or JMJD1A) were also described in BC [76]. Furthermore, a strong H3K9ac signal in promoters of specific genes (such as *FGF14*, *PAX3*, *DLX5*, *DLX6*, *MYT1*, *HAND2*, *GATA4*) was observed. H3K27me3 enrichment on the *RUNX1* gene was determined in HER2-positive and Luminal-A subtypes but was down-regulated in tumors with luminal-B1 and -B2 subtypes. Moreover, *RUNX1* expression correlated with the poorest prognosis, likely due to its action in diminishing ER signaling [77]. Reduced levels of H3K9me2 epi-marks in primary breast epithelial cells were found involved in up-regulation of PAX3 expression in HER2-positive tumors [76]. Finally, low levels of H4R3me2, H3K9ac, and H4K16ac were significantly associated with large tumor size, while enrichment of H4R3me2 and H3K9ac were associated with low lymph node stage [73].

Specific miRNA signatures have been correlated with tumor aggressiveness, drug response, and patient outcome in BC. Various studies demonstrated that BC subtypes exhibit different miRNA signatures [78–82] (Table 2). Some miRNAs (miR-520g, miR-377, miR-527- 518a, miR-29, miR-513a-5p, and miR-520f-520c) are able to influence PR status in BC [83,84]. A mechanism by which progesterone induces loss of miR-141 and BC de-differentiation deregulating PR and Stat5a was proposed [85]. It was suggested that up-regulation of miR-342 expression in ER-positive/HER2-positive BC influences ER expression and response to tamoxifen [83,86]. Furthermore, up-regulation of the exosomal miR-221/222 cluster was associated with tamoxifen resistance in luminal-type BC cells by direct targeting of p27kip1 [87]. Overexpression of miR-221 in BC was identified as a good prognosis marker and is associated with ER positivity and lymph node negativity [88]. Additionally, miR-210 and miR-21 were found potentially useful for predicting and/or monitoring response to trastuzumab [89]. Finally, analysis of miRNA expression levels by NMF consensus clustering of miRNA-seq abundance profiles for 697 tumor samples identified seven subtypes correlating with mRNA subtypes based on ER, PR, and HER2 clinical status. Notably, two miRNA groups overlap with the basal-like mRNA subtype and include many miRNA signature mutations. The other miRNA groups are made up of a combination of luminal-A/B and HER2-positive tumors, presenting little correlation with the subtypes previously identified using the differential expression of 50 genes (PAM50) [90].

**Table 2:** The microRNAs associated with molecular subtypes of BC.

Clinical subtypes	Overexpression	Underexpression
Luminal A	let-7c, let-7f miR-10a miR-191 miR-26 miR-190b miR-99a miR-130 miR-126 miR-136 miR-146b miR-100	miR-206 miR-15b miR-107 miR-103
Luminal B	miR-342 miR-15b miR-107 miR-103	miR-100 miR-99a miR-130 miR-126 miR-136
HER2-enriched	miR-142-3p miR-150	miR-125a/b
Triple negative	miR-18a/b miR-135b miR-93 miR-155 miR-17-92 miR-10b miR-26a miR-153	miR-29 miR-190b

However, molecular profiling studies do not reflect the heterogeneity of constituent cell types and their interactions within tumor microenvironment. Onuchic and colleagues developed an in silico deconvolution technique (EDec) that estimates cell type composition, as well as DNA methylation and RNA profiles of constituent cell types within BCs: cancerous epithelial, normal epithelial, and stromal/immune fractions [91]. The EDec method deconvoluted molecular profiles of BCs within the TCGA collection (http://genboree.org/theCommons/projects/edec). Specifically, EDec recognizes different combinations of the five cancerous epithelial components within each tissue sample, identifying one predominant component establishing the specific subtype. Grouping samples based on the predominant component displayed some concordance with their PAM50 classification [90]. Luminal-B tumors showed the most heterogeneous profiles and normal breast samples the most homogeneous epithelial profile, whereas basal-like tumors presented epithelial methylation profiles highly distinct from the other BC subtypes. The degree of immune/stromal cell infiltration across the tumor was also different, with the highest and lowest values in basal-like and luminal-B cancers, respectively. The EDec method confirmed the association between increased immune cell infiltration and better prognosis [92]; patients with ≥20% estimated immune cell type proportion survived longer than those with ≤20%. Moreover, by comparing gene expression profiles of tumor samples against normal control counterparts, EDec found enriched changes associable with known cancer hallmarks and known roles of each BC cell type [21]. Lastly, the authors found gene enrichment in tumor stroma for oxidative phosphorylation (OXPHOS) and glycolysis among down- and up-regulated genes, respectively. OXPHOS gene down-regulation reflects the change in stromal composition from a more adipose (oxidative) stroma in normal breast to a more fibrous (glycolytic) stroma in tumors, supporting the oxidative metabolism of BC. Stromal composition is therefore able to predict metabolic coupling between epithelial and stromal cancer cells.

The role of tumor-infiltrating regulatory T-lymphocytes (Tregs) in this context is also of note. Tregs are fundamental in cancer homeostasis and are associated with cancer progression and clinical outcome [93]. These cells block antitumor T-cell response by coordinated activation of immune checkpoints in infiltrating leukocytes and lymphocytes [94,95]. Multiple clinical trials with molecules able to block immune checkpoints showed encouraging results in cancer therapy [96,97]. Hence, a better molecular characterization of Treg biology may help develop more effective therapeutic strategies. Plitas and colleagues performed transcriptome analysis of human Tregs isolated from untreated human BC, normal mammary tissue, and peripheral blood samples [98]. Tregs were variably present in BC, reaching the highest frequency in more aggressive tumors, such as TNBC. Tumor-resident Tregs displayed very similar expression patterns to those of Tregs resident in normal parenchyma, but different from those of activated peripheral blood Tregs, resulting mainly in enrichments in GO terms for cytokine signaling, defense, and inflammatory response. The chemokine receptor CCR8 resulted overexpressed in tumor Tregs and represents a novel target in immunotherapeutic approaches for BC treatment. CCR8 induction was obtained by co-culturing activated peripheral blood Treg cells with tumor explants, indicating the crucial role of tumor microenvironment. CCR8 was found overexpressed in other cancer types, including lung and colorectal adenocarcinomas, melanoma, and angiosarcoma [98]. These data were independently corroborated by De Simone and colleagues, who defined a subset of signature genes in Tregs infiltrating non-small-cell lung cancer (NSCLC) and colorectal cancer (CRC) [99]. In whole-tumor samples, overexpression of *LAYN*, *MAGEH1*, and *CCR8*, three of the most enriched genes in tumor-infiltrating Treg signature genes, was associated with a worse 5-year survival of CRC and NSCLC patients, pinpointing these genes as potential therapeutic targets. These promising data suggest that transcriptome analysis of lymphocytes from different cancer types could improve our understanding of the dynamics of microenvironment-associated immune modulation, which is crucial to identify novel therapeutic targets able to reduce selectively tumor-infiltrating Tregs.

In support of the impact of epigenome influence on BC, inhibition of PI3K pathway was reported to lead to activation of ER-dependent transcription through the epigenetic regulator KMT2D, identified as the key determinant [100]. In mice, genetically removing KMT2D and inhibiting PI3K pathway achieved higher tumor shrinkage than either therapy alone. These findings provide a rationale for epigenetic therapy in patients with PIK3CA-mutant, ER-positive BC [100].

Overall, recent findings highlight that epigenomic and transcriptomic profiles of BC cells are highly distinct across different subgroups, as well as within the same subtype, and that a more detailed deconvolution of individual cellular components could provide important prognostic and therapeutic insights. In addition, integrated epigenome and genome characterization may provide therapeutically exploitable explanations for metastatic BC. To date, several clinical trials have assessed the effects of HDACi alone or in various combinations (Table 3). Promising results were obtained from combinations with endocrine therapy involving vorinostat and entinostat, due to their potential role in reverting tamoxifen resistance [101] (NCT01194427/NCT00365599) and reverting aromatase inhibitor resistance (NCT00828854/NCT02820961), respectively. Combining HDACi with chemotherapy in treating women with newly diagnosed and metastatic BC has also provided good results (NCT00616967/NCT00368875/NCT01010854). Trials of HDACi (vorinostat/panobinostat) in combination with HER2-targeted therapy (trastuzumab) have been encouraging (NCT00258349/NCT00567879). Indeed, the FDA very recently designated Entinostat as a “breakthrough therapy for the treatment of locally recurrent or metastatic ER-positive BC, when added to exemestane in post-menopausal women whose disease has progressed following non-steroidal aromatase inhibitor therapy,” based on data from the completed phase II ENCORE 301 study [102].

**Table 3:** Clinical trials for breast cancer.

NCT number	Clinical trial	Condition	Drug	Phase	Sponsor
NCT01194427	A study of vorinostat and tamoxifen in newly diagnosed breast cancer	Stage I breast cancer Stage II breast cancer Stage III breast cancer Invasive breast cancer	Vorinostat and tamoxifen	2	Sidney Kimmel Comprehensive Cancer Center
NCT00365599	Phase II trial of SAHA and tamoxifen for patients with breast cancer	Breast cancer	Vorinostat and tamoxifen	2	H. Lee Moffitt Cancer Center and Research Institute
NCT00828854	A phase II, multicenter study of the effect of the addition of SNDX-275 to continued aromatase inhibitor (AI) therapy in postmenopausal women with ER-positive breast cancer whose disease is progressing	Estrogen receptor-positive breast cancer	Entinostat	2	Syndax Pharmaceuticals
NCT02820961	Drug-drug interaction study of entinostat and exemestane in postmenopausal women with ER-positive breast cancer	Breast cancer Estrogen receptor-positive breast cancer	Entinostat and exemestane	1	Syndax Pharmaceuticals
NCT00616967	Carboplatin and nab-paclitaxel with or without vorinostat in treating women with newly diagnosed operable breast cancer	Breast cancer	Carboplatin, Paclitaxel albumin-stabilized nanoparticle formulation, Vorinostat	2	Sidney Kimmel Comprehensive Cancer Center
NCT00368875	Study phase I-II study of vorinostat, paclitaxel, and bevacizumab in metastatic breast cancer	Male breast cancer Stage IIIB breast cancer Stage IIIC breast cancer Stage IV breast cancerr	Vorinostat and paclitaxel	1/2	National Cancer Institute
NCT01010854	Valproic acid in combination with FEC100 for primary therapy in patients with breast cancer (VPA-FEC100)	Breast cancer	VPA FEC100	2	University of California, San Francisco
NCT00258349	Vorinostat and trastuzumab in treating patients with metastatic or locally recurrent breast cancer	Breast cancer Male breast cancer Recurrent breast cancer Stage IIIB breast cancer Stage IIIC breast cancerr Stage IV breast cancer	Vorinostat and trastuzumab	1/2	National Cancer Institute
NCT00567879	A trial of panobinostat and trastuzumab for adult female patients with HER2-positive metastatic breast cancer whose disease has progressed on or after trastuzumab	Breast cancer	Panobinostat and trastuzumab	1/2	Novartis Pharmaceuticals

### Rhabdoid tumor

Malignant rhabdoid tumor (MRT) is a rare pediatric soft tissue sarcoma that can arise in any part of the body, but most commonly occurs in the brain, where it is called an atypical teratoid/rhadboid tumor (AT/RT) [103]. Excluding rare cases resulting from loss of *SMARCA4* [104–106], AT/RT is mainly driven by loss of *SMARCB1* [107] associated with aberrations of chromosome band 22q11.2 [107,108] in restricted neural precursors. SMARCB1 and SMARCA4 are members of chromatin remodeling SWI/SNF complex [108]. Molecular studies have elucidated the mechanism by which absence of this TSG promotes cancer. *SMARCB1* knockout mice models indicated its critical role in preventing cancer [109,110]. *SMARCB1* loss induces (i) cell cycle progression via *p16/INK4A* down-regulation and *cyclin D1* and *E2F* up-regulation [111–115], (ii) aberrant activation of Hedgehog-Gli pathway [116] and β-catenin/TCF targets [117], (iii) repression of neural development [118], and (iv) elevated expression of *EZH2* leading to increased levels of H3K27me3 at lineage-specific polycomb targets [119], as demonstrated by the use of tazemetostat, able to induce apoptosis and differentiation in *SMARCB1*-deleted MRT cells [120]. Two clinical trials are investigating tazemetostat [121] in patients with genetically defined solid tumors, including *SMARCB1/INI1*-negative tumors (NCT02601937/NCT02601950) or any solid tumor with EZH2 gain-of function mutation (NCT02601950) (Table 4). Despite therapeutic advances, AT/RT prognosis is very poor. Some patients do respond positively to standard treatment, indicating that molecular heterogeneity among individuals is responsible for the clinical range of AT/RTs. However, an increasing number of genomic analyses identified *SMARCB1* loss as the recurrent genetic event in AT/RT [122,123]. Investigating the correlation between molecular heterogeneity and determinants of survival and therapeutic response is hampered by the rare incidence of AT/RT. To clarify the molecular basis for AT/RT clinical heterogeneity, Torchia and colleagues performed an integrated molecular and clinicopathological analysis, identifying two molecularly distinct subgroups and three separate risk categories [124]. This important study opened the way towards a novel integrated, risk-adapted therapeutic approach for AT/RTs.

**Table 4:** Clinical trials for rhabdoid tumor.

NCT number	Clinical trial	Condition	Drug	Phase	Sponsor
NCT02601937	A phase I study of the EZH2 inhibitor tazemetostat in pediatric subjects with relapsed or refractory INI1-negative tumors or synovial sarcoma	Rhabdoid tumors INI1-negative tumors Synovial sarcoma Malignant rhabdoid tumor of ovary Stage II breast cancer Stage III breast cancer Invasive breast cancer	Tazemetostat	1	Epizyme
NCT02601950	A phase II, multicenter study of the EZH2 inhibitor tazemetostat in adult subjects with INI1-negative tumors or relapsed/refractory synovial sarcoma	Malignant rhabdoid tumors Rhabdoid tumors of the kidney Atypical teratoid rhabdoid tumors Selected tumors with rhabdoid features Synovial sarcoma INI1-negative tumors Malignant rhabdoid tumor of ovary Renal medullary carcinoma Epithelioid sarcoma Any solid tumor with an EZH2 GOF mutation	Tazemetostat	2	Epizyme

Johann and colleagues recently investigated the transcriptomic and epigenomic landscape of primary AT/RT samples [125]. DNA methylation and transcriptional profiles of 192 primary AT/RTs identified three molecular subgroups, named AT/RT-TYR, -MYC, and -SHH, all showing *SMARCB1* alterations but different tumor location caused by the different nature of precursor cells. Genes frequently overexpressed are members of PRC2 (EZH2, SUZ12, EED), validating and extending the previously reported prognostic and therapeutic importance of the complex. This classification highlights how high inter-tumor heterogeneity may derive from differences in epigenetic profiles. A complete WGBS analysis comparing 17 AT/RT cases with other pediatric brain tumors and normal controls showed that AT/RT-TYR and -SHH subgroups exhibited a more hypermethylated genome. Hypermethylation was mostly found in promoter regions and in AT/RT-SHH. Several TSGs, including *GLIPR1*, showed promoter hypermethylation and resulted silenced in AT/RT-TYR and AT/RT-SHH. Furthermore, partially methylated domains explained differences in both global methylation and transcriptional profiles in the three subgroups. Genome-wide H3K27Ac and Bromodomain-containing protein 4 (BRD4) ChIP sequencing showed that DNA methylation did not correlate with H3K27ac signal, while BRD4 and H3K27Ac were highly correlated. DNA methylation valleys [126] were highly enriched for both of these marks, overlapping with the majority of enhancers. Comparing all AT/RT enhancers called in H3K27Ac ChIP with those identified by ENCODE and Roadmap [10,127], 11.5% were found unique to AT/RTs. In an attempt to correlate transcriptional differences between the three subgroups with group-specific subsets of active enhancers, the authors classified differently regulated enhancers into four distinct clusters: TYR-specific (the most abundant), MYC-specific, TYR_SHH high, and SHH-specific. These subgroup-specific enhancers include super-enhancers, important for cellular identity and identifying the master gene regulators of each subgroup [128,129]. Analyzing enrichment of TF binding sites within subgroup-specific enhancers identified a set of specific TFs for each AT/RT subgroup. Specifically, enriched and overexpressed TFs in each group were OTX2 and LMX1A in AT/RT-TYR; SHH effectors such as GLI2 and FOXK1 in AT/RT-SHH; and RARG, CEBPB, and MYC in AT/RT-MYC.

This study represents a milestone in our understanding of AT/RT biology. The regulatory networks and TFs identified as drivers of each specific subgroup (such as MITF in AT/RT-TYR) may provide novel targets in the search for more effective drugs in molecularly based therapies.

### Risks and bottlenecks in cancer epigenetics

Despite groundbreaking findings, bottlenecks may slow down advances in our knowledge of cancer epigenetics. As the knowledge of chromatin remodeling and modification is constantly expanding, our current view is, by definition, limited. Scientific advancement is bound to technological developments; progress is linked to new applied techniques, such as single-cell analysis applied to transcriptome [130–132] and chromatin regulations [133,134] in the study of tumor heterogeneity. Consequently, novel technological developments are likely to profoundly modify our understanding of the role, and data mining, of cancer epigenomes. The increasing need, development, and use of bioinformatics (and dedicated analyses) also underscores the huge change in our way of dealing with human health and identifying predisposition, diagnosis, and treatment. Furthermore, the lesson learned from hydroxymethylcytosine and DNA methylation regulation [135–137] (referring to the evidence that a cytosine needs to be methylated before being hydroxymethylated) suggests that prioritization of sets of modifications should be taken into account and potentially integrated to obtain a more complete, potentially multi-step landscape of the epigenome and its deregulation in cancer. From this perspective, decoding the role of spatial-temporal regulation of chromatin in cancer is currently limited by both technical and analytical issues. New insights will lead to a more complete understanding of tumorigenesis, defining dynamics, 3D heterogeneity, and necessary versus sufficient steps of cancerogenesis and metastatic progression.

Thus, our understanding of cancer epigenomes and carcinogenesis will evolve, casting new light on cancer progression steps and potential interventions [138]. For example, linking large-scale epigenomic profiles to expression quantitative trait loci (eQTLs) allowed prediction of critical chromatin features associated with varying regulatory potential [139]. Using phenotypically relevant epigenomes to weight GWAS single-nucleotide polymorphisms improved the statistical power of gene-based association [140]. Further disease-oriented validations and dedicated selective epi-based approaches are required.

Although considerable progress has been made in determining the composition and functionalization of specific protein complexes, studies have mostly investigated isolated protein components in different cellular systems. A systematic proteomic approach (and its integration with other omic approaches) will make it possible to comprehensively map complexes and factors on chromatin and identify new interactors. Determining which subunits are responsible for complex tethering to chromatin may provide new insights into molecular function and complex recruitment. Considering the enormous diversity among individual complexes, such an approach could provide novel hypotheses for experiments aimed at disentangling the biochemistry of specific protein-mediated gene regulation in healthy and tumor cells to unequivocally define specific groups of normal and cancer cells.

Finally, postulating the parallel advancement of both science and technology, one main risk in both cancer and healthy epigenome evaluation is the possibility of patient re-identification, opening up ethical issues and additional practical concerns [141].

Personalized medicine and healthcare following a bottom-up strategy is a highly desirable goal. Consequently, all major grant agencies around the world dedicate funding to integrate omic studies into the clinic, even if limitations on the use of patient data in health services still exist [142].

Translating the advances of epigenome deregulation to the clinic may require a much better understanding of the multiple challenges in developing epidrugs [143]. For example, the lack of locus-selective specificity may create (epi)genome-wide “off- target” effects. This potential drawback of epidrugs might be resolved by using new “epigenome editing” approaches [144]. On the other hand, novel epidrugs directed to genetically mutated chromatin players might represent a different, more focused solution. The development of novel drug design approaches (such as the proteolysis targeting chimera (PROTAC) approach applied to epi-targeting chemical scaffolds) might also lead to the development of specific epidrugs against non-enzymatic chromatin players [145]. Rapid advances in our knowledge of the mechanisms of action of chromatin complexes in (epi)genome control, allowing localized detections and epigenome fine-tuning, might make it possible to correct or exploit susceptibilities of epigenetically deregulated cells, complementing or potentially transcending cancer genetics.

## Conclusions

Unraveling epigenetic and genetic changes involved in cancer pathogenesis has always been, and to a large extent still is, a huge task. We are currently gaining greater insights into how the epigenome determines cell type specification, development, and pathology. IHEC results have shed new light on molecular mechanisms of disease, highlighting the fundamental challenges in understanding causal networks between genotype and phenotype. Epigenome profiles are also able to define distinct cellular identities and specific cell–cell interactions driving tumorigenesis. Acquired knowledge and its exploitation in preclinical and clinical settings for hematological malignancies, as well as common adult solid cancers (such as BC) and pediatric tumors (including RT), is just an example of the headway made in cancer epigenetics. There is still an urgent need to translate the findings to the clinic, where they may be used for diagnostic, prognostic, and treatment response evaluation. While for some cancers (such as those described here), this is already (or just one step away from) reality, for others further developments are still required.

Thus, understanding the functional outcome of complex cancer-associated epigenetic variations at molecular level is critical. The clearest effect of such modifications is on gene expression. However, it is also crucial to gain a greater insight into how epigenetic modifications alter the structure of (neighboring) chromatin and the binding of key TFs and whether and how they are associated with or instigate other epigenetic changes, such as histone modifications. Comparing epigenetic with GWAS data identified variants individually associated with disease risk [146,147]. Herein lies the unprecedented advances made by IHEC, which has generated and disseminated reference maps of human epigenomes for pivotal cellular states involved in health and disease.

**Fig 2:**
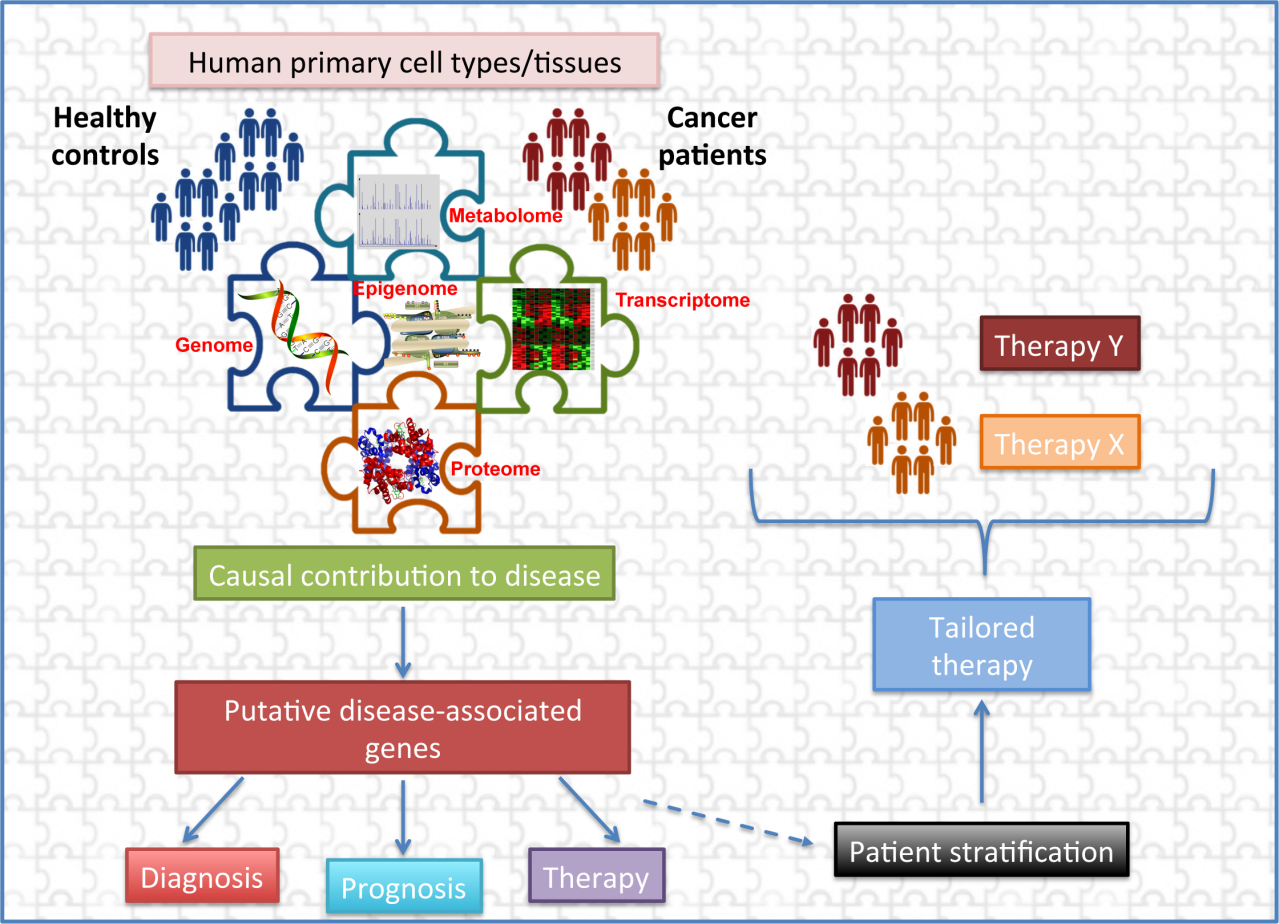
Integrated genome-wide analysis. Integrating and combining data from different platforms (genome-DNA sequence, transcriptome, proteome, metabolome, and epigenome) leads to a better understanding of the basis of cancer and paves the way toward personalized medicine.

Overcoming (some of) the above limitations, together with advances in scientific research, will pave the way toward truly personalized therapies and clinical applications in diagnosis, prognosis, and prevention, which will represent a major step forward in cancer epigenetics (Fig 2). Our understanding of how chromatin architecture, DNA methylation, and gene expression impacts cancer is changing our views on cancerogenesis, as well as on cancer staging and classification. Epigenetic modifications could become useful biomarkers for prognosis and treatment of disease, further shifting the focus of epi-data from bench to bedside.
